# Evaluating Microglial Dysfunction and Psychiatric Illness by Exploring the Inflammatory Basis of Schizophrenia and Depression in Cross-Sectional Study settings

**DOI:** 10.7759/cureus.82831

**Published:** 2025-04-23

**Authors:** S Jamil Hussain, Noor-e-Hira Memon, Fahad ul Zain, Syed Muhammad Shahkar Ali, Saifullah Mahar, Anil Kumar Wadhwani, Muhammad Haseeb, Muhmmad Hussain Shah

**Affiliations:** 1 Department of Psychiatry, The University of Modern Sciences, Tando Muhammad Khan, NawabShah, PAK; 2 Department of Psychiatry, Black Country Healthcare NHS Foundation Trust, Wolverhampton, GBR; 3 Department of Psychiatry, People's University of Medical and Health Sciences, NawabShah, PAK; 4 Department of Psychiatry and Behavioural Sciences, Mufti Mehmood Memorial Teaching Hospital, Dera Ismail Khan, PAK; 5 Nursing, Begum Bilqees Sultana Institute of Nursing, NawabShah, PAK; 6 Department of Psychiatry, Jinnah Sindh Medical University, Karachi, PAK; 7 Oncology, Shaikh Zayed Hospital, Lahore, PAK; 8 Pathology, Dow University of Health Sciences, Dow International Medical College, Karachi, PAK

**Keywords:** cytokines, depression, microglia, neuroimmune interaction, neuroinflammation, psychiatric biomarkers, schizophrenia

## Abstract

Background: Growing evidence in research demonstrates that neuroinflammation plays an important role in the pathophysiology of schizophrenia and major depressive disorder. This study evaluated how peripheral inflammatory cytokines relate to the intensity of the psychiatric symptoms of these disorders.

Methods: A cross-sectional study was conducted that included 100 participants, of which 30 were schizophrenia patients, 30 were major depressive disorder (MDD) patients, and 40 were healthy controls. The inflammatory markers, also known as peripheral cytokines IL-6, TNF-α, and IL-1β, were measured using an ELISA method. The Positive and Negative Syndrome Scale (PANSS) and the Hamilton Depression Rating Scale (HAM-D) tools were used to evaluate symptom severity. To perform group comparisons, ANOVA was used, and for correlations, Pearson’s method was used.

Results: TNF-α and the cytokines IL-6 and IL-1β were found to be higher in patients than in control groups (p < 0.001), where the highest levels were observed in schizophrenia patients. PANSS scores in schizophrenia patients correlated with IL-6 and TNF-α serum levels, whereas MDD patients showed correlations between IL-6 and IL-1β levels and HAM-D scores.

Conclusion: Peripheral inflammatory markers were seen to be associated with psychiatric symptom severity, which supported the inflammatory hypothesis of mental illness. These cytokines might serve as accessible biomarkers. However, direct microglial assessment and longitudinal studies were needed for confirmation of results.

## Introduction

Medical experts have gained an understanding of the essential role of neuroinflammation in major psychiatric diseases because evidence showed defects in microglial cells during schizophrenia and major depressive disorder (MDD) [1]. In the central nervous system, microglial cells maintain their identity as immune cells while they control the developmental pathways, synaptic connections, and inflammatory reactions [2,3]. These cells change their phenotype to become pro-inflammatory when confronted with persistent stress or pathology, which causes them to release cytokines that damage neural functions and produce behavioral problems [4].

According to the research studies, traditional neurotransmitter-based illness models differ from the neuroimmune framework because they demonstrate immune dysregulation as the foundation for psychiatric symptom development and maintenance [5]. Available research demonstrates that schizophrenia and depression both have shown increased rates of cytokines, including IL-1β, IL-6, and TNF-α, which indicate systemic inflammation followed by cognitive impairment, treatment unresponsiveness, and disturbances in mood [6]. Present research primarily studied individual inflammatory markers, but the comprehensive study on microglial activation processes and resulting consequences in various psychiatric populations has been done only to a limited extent [7]. Peripheral cytokines became the primary focus because they are widely available and scalable in psychiatric care, particularly in low-resource settings. Microglia evaluation remained unapproachable for practical use in large-scale clinical trials and low-income regions. This study connected translational research by identifying measurable biomarkers that allowed for the development of anti-inflammatory treatment strategies, but still, clinical utility is required for additional multi-modal validation [8].

This research analyzed how microglial-linked inflammatory cytokines are connected to specific psychiatric symptoms in schizophrenia and depression. The evaluation of biological markers with clinical symptoms in affected patients aimed to advance our knowledge about inflammatory mechanisms during psychiatric illnesses. Research demonstrated the translational worth of microglial activity manipulation, which serves as a base for developing immune-based psychiatric therapies.

## Materials and methods

A cross-sectional investigation took place between January 2024 and July 2024 at psychiatric outpatient departments within Pakistan. The Indus Medical College Hospital issued approval (040325-45-MS). Determination of sample size was conducted using an Open API version 3.0.0 that recommended a total sample of 100 participants (60 patients and 40 healthy controls) to achieve the 80% power at a 0.05 significance level while assuming a moderate effect size (Cohen’s d = 0.5). Patients were classified into two disease groups: 30 with schizophrenia and 30 with. Controls were age and gender matched individuals who didn’t have any psychiatric or inflammatory comorbidities. Clinical diagnosis occurred through interviews conducted by psychiatrists aligned with DSM-5 standards. The participants signed written consents that authorized their involvement in the study. Participants needed to fit the following requirements for inclusion: Adults aged 18-50 years, DSM-5 diagnosis of schizophrenia or MDD (for patient groups), stable medication regimen for ≥6 weeks (if applicable), and no comorbid neurological, autoimmune, or infectious diseases.

The study excluded patients who had: a history of traumatic brain injury, substance abuse, or non-psychiatric systemic illnesses, used immunomodulatory drugs (e.g., steroids, nonsteroidal anti-inflammatory drugs (NSAIDs)) within the past 3 months, and healthy controls with any lifetime psychiatric diagnosis. The research team performed complete assessments on each subject through the Positive and Negative Syndrome Scale (PANSS) for schizophrenia and the Hamilton Depression Rating Scale (HAM-D) for MDD symptom evaluation, together with demographic surveys (including age, gender, and other socioeconomic indicators). While fasting in early mornings, researchers measured pro-inflammatory cytokines: IL-6, TNF-α, and IL-1β through ELISA tests for blood serum collected from each participant. SPSS version 25 was used to analyze the data collection. Descriptive statistics were employed to summarize baseline characteristics. All groups underwent independent t-tests to measure cytokine differences, but Pearson’s correlation method analyzed the connection between cytokine levels and symptom severity scores. The authors used one-way ANOVA with Tukey’s post-hoc test to study comparisons between groups with a statistically significant threshold at a p-value lower than 0.05.

## Results

A research sample of 100 participants was taken that consisted of 30 individuals who received a diagnosis of schizophrenia and 30 patients who received a diagnosis of MDD, while 40 were controls. Participant in the study sample had an average age of 32.2 years with a standard deviation of 7.8 years. Table 1 presents the demographic distribution. Female participants represented 64% of the MDD group, but schizophrenia participants consisted of 63% males.

**Table 1:** Demographic Characteristics of Participants MDD: major depressive disorder

Variable	Schizophrenia (n=30)	MDD (n=30)	Controls (n=40)	p-value
Age (years)	32.4 ± 7.8	31.1 ± 8.2	33.0 ± 7.5	0.62
Male (%)	63%	36%	55%	0.08
Female (%)	37%	64%	45%	0.08
BMI (kg/m²)	26.5 ± 4.1	27.2 ± 4.8	25.8 ± 3.9	0.34
Antipsychotic use (%)	87%	13%	0%	<0.001
Antidepressant use (%)	20%	83%	0%	<0.001
Illness duration (y)	5.1 ± 2.9	4.7 ± 3.2	N/A	0.58
Urban residence (%)	70%	66%	75%	0.68
Low socioeconomic status (%)	58%	60%	45%	0.84

Biomarkers responsible for inflammation reached statistically significant elevations, and schizophrenia participants maintained higher biomarker levels than other groups. The samples from schizophrenia patients and MDD patients demonstrated elevated IL-6 with TNF-α, and IL-1β against control group values, as shown in Table 2.

**Table 2:** Inflammatory Cytokine Levels Across Study Groups MDD: major depressive disorder

Biomarker	Schizophrenia (n=30)	MDD (n=30)	Controls (n=40)	Test Statistic	p-value
IL-6 (pg/mL)	18.9 ± 4.3	14.6 ± 3.7	3.2 ± 1.1	F = 25.7	<0.001*
TNF-α (pg/mL)	25.5 ± 5.0	20.9 ± 4.5	6.8 ± 2.3	F = 18.4	<0.001*
IL-1β (pg/mL)	12.3 ± 2.6	9.8 ± 2.2	1.5 ± 0.7	F = 30.1	<0.001*

The researchers found that psychiatric symptom intensity had a strong positive relationship with detected cytokine levels. The PANSS total scores in schizophrenia displayed moderate-to-strong relations with IL-6 and TNF-α (r = 0.54 and r = 0.48), yet IL-6 and IL-1β exhibited these relations with HAM-D in patients with MDD (r = 0.46 and r = 0.41). Table 3 summarizes the correlation values.

**Table 3:** Correlation Between Cytokines and Symptom Severity MDD: major depressive disorder; PANSS: Positive and Negative Syndrome Scale

Group	Biomarker	Scale	Correlation (r)	p-value
Schizophrenia	IL-6	PANSS	0.54	<0.01
Schizophrenia	TNF-α	PANSS	0.48	<0.01
MDD	IL-6	HAM-D	0.46	0.01
MDD	IL-1β	HAM-D	0.41	0.02

These results confirm a significant inflammatory component in both psychiatric disorders, with schizophrenia showing more pronounced microglial biomarker elevation.

## Discussion

The study evaluated the peripheral pro-inflammatory cytokines in relation to psychiatric symptom severity in patients with schizophrenia and MDD. Research indicated that schizophrenia and MDD patients had higher levels of IL-6, TNF-α, and IL-1β as compared to healthy controls. These experimental results aligned with the documented evidence of inflammation as an important factor in the development of psychiatric disorders, particularly schizophrenia [9].

The associations between the elevated cytokine levels and clinical symptom scores further supported the neuroinflammatory framework. In schizophrenia, IL-6 and TNF-α were positively correlated with PANSS scores while indicating their involvement in symptom severity [10]. IL-6 is believed to impair synaptic plasticity and disrupt neurotransmission, while TNF-α may interfere with glial-neuronal communication, both of which were consistent with mechanisms linked to microglial activation. In MDD, IL-6 and IL-1β showed moderate correlations with HAM-D scores. This correlation supported the cytokine hypothesis of depression in which immune signaling was thought to impact serotonin pathways, hypothalamic-pituitary-adrenal (HPA) axis, and neuroplasticity [11,12].

The findings established that microglial activation-induced immune dysregulation played a role in creating psychotic symptoms. The inflammatory cytokine levels in MDD patients remained lower than schizophrenia, yet IL-6 and IL-1β showed significant links to depression intensity. The “cytokine hypothesis of depression” received support from these research findings, which showed that peripheral and central immune signaling mechanisms affect mood regulation by potentially causing reduced serotonin availability and dysfunction in both the HPA axis and the processes of neuroplasticity [13]. The research identified that inflammation is caused regardless of gender and socioeconomic factors when examining brain cytokine concentration. Research findings confirm the presence of male prevalence in schizophrenia, while female patients exceed men in MDD cases because of potential hormonal and psychosocial influences on inflammation.

Scientists have validated the concept of interspersed yet separate inflammatory processes that link schizophrenia to depression [14,15]. Schizophrenic inflammation appears to be more severe than depression, but this disorder also exhibits important inflammatory pathways. Research supports the development of psychiatric care approaches that unite immunological assessment together with therapeutic approaches [16].

The study design restricts any potential conclusions about cause-and-effect relationships, and cytokine measurements from blood may differ from those found in brain tissue inflammation. The study controlled neither the drug medication impact on cytokine levels nor was able to generalize results beyond limitations in sample size. Furthermore, lifestyle factors such as diet, smoking, or sleep quality were not evaluated, which might have influenced systemic inflammation. Future psychiatric research should use longitudinal study designs and microglial cellular measurement techniques via translocator protein (TSPO) ligand positron emission tomography (PET) scans and must evaluate anti-inflammatory drug effects in treatment procedures.

## Conclusions

This study demonstrated that schizophrenia and MDD were inflammatory disorders because enhanced IL-6, TNF-α, and IL-1β levels showed direct links to symptom intensity in affected patients. The higher levels of inflammation observed in schizophrenia patients indicated that microglial dysfunction played a significant role in schizophrenia development than in depression cases. The results emphasized the crucial need to use neuroimmune biomarkers both in psychiatric diagnostic approaches and decision-making in treatment development.

Discovering patterns of psychiatric disorders against inflammation would lead to better intervention strategies, especially when emphasizing neuroinflammation treatments. The obtained information presented opportunities to advance both immunopsychiatric research and clinical mental health treatment effectiveness.
